# Current and Future Costs of Intractable Conflicts—Can They Create Attitude Change?

**DOI:** 10.3389/fpsyg.2021.681883

**Published:** 2021-05-26

**Authors:** Nimrod Rosler, Boaz Hameiri, Daniel Bar-Tal, Dalia Christophe, Sigal Azaria-Tamir

**Affiliations:** ^1^Program in Conflict Resolution and Mediation, Tel Aviv University, Tel Aviv, Israel; ^2^School of Education, Tel Aviv University, Tel Aviv, Israel

**Keywords:** cost, intractable conflicts, democracy, Israeli-Palestinian conflict, openess

## Abstract

Members of societies involved in an intractable conflict usually consider costs that stem from the continuation of the conflict as unavoidable and even justify for their collective existence. This perception is well-anchored in widely shared conflict-supporting narratives that motivate them to avoid information that challenges their views about the conflict. However, since providing information about such major costs as a method for moderating conflict-related views has not been receiving much attention, in this research, we explore this venue. We examine what kind of costs, and under what conditions, exposure to major costs of a conflict affects openness to information and conciliatory attitudes among Israeli Jews in the context of the intractable Israeli–Palestinian conflict. Study 1 (*N* = 255) revealed that interventions based on messages providing information on mental health cost, economic cost, and cost of the conflict to Israeli democracy had (almost) no significant effect on perceptions of the participants of these prices, openness to new information about the conflict, or support for conciliatory policies. However, the existing perceptions that participants had about the cost of the conflict to Israeli democracy were positively associated with openness to alternative information about the conflict and support for conciliatory policies. Therefore, in Study 2 (*N* = 255), we tested whether providing information about future potential costs to the two fundamental characteristics of Israel, a democracy or a Jewish state, created by the continuation of the conflict, will induce attitude change regarding the conflict. The results indicate that information on the future cost to the democratic identity of Israel significantly affected the attitude of the participants regarding the conflict, while the effect was moderated by the level of religiosity. For secular participants, this manipulation created more openness to alternative information about the conflict and increased support for conciliatory policies, but for religious participants, it backfired. We discuss implications for the role of information about losses and the relationship between religiosity and attitudes regarding democracy and conflict.

## Introduction

Societies that live under the harsh circumstances of a prolonged, violent conflict invest huge material and psychological resources for their continuous management (Kriesberg, 1993). Most probably, an outsider to the conflict could easily identify such investments as costs that can be eliminated, or at least mitigated, by ending the conflict peacefully. However, members of societies involved in the conflict usually consider their investment in the conflict as unavoidable and even justify and praise it as a necessary sacrifice for their collective existence (Bar-Tal, 2013). This perception is well-anchored in their collective conflict-supporting narratives that provide explanation for the outbreak of the conflict, justify its continuation, and provide rationale for the required immense investments. The conflict-supporting narratives are developed to enable society members to cope with the difficulties encountered and to keep ongoing mobilization for the persistence of the conflict. Thus, the narratives serve as a barrier to ideas and policies aimed at resolving the conflict peacefully (see Cairns and Roe, 2003; Coleman et al., 2009; Halperin and Bar-Tal, 2011; Garagozov, 2012).

The research indicates that large portions of individuals who live in societies immersed in intractable conflicts go through routinization. This process gives the abnormality of life, in the context of such conflict, a sense of normalcy, which includes ignoring hardships, stress, and other negative consequences (Bar-Tal et al., 2014). Society members, in this context, oftentimes prefer closure over free flow of information that can challenge their worldviews and perceptions about the conflict, the enemy, and the in-group (e.g., De Zavala et al., 2010; Hameiri et al., 2017). In fact, in these contexts, individuals are often driven by the motivation to be exposed to a specific content that confirms their held beliefs and attitudes and to avoid information that challenges their views (Kruglanski, 2004). Thus, they are likely to avoid information about the costs their society is paying for the continuation of the conflict; and even when exposed, they are likely to reject it using various defense mechanisms (e.g., Kunda, 1990; Kruglanski, 2004).

Nevertheless, this premise cannot be generalized to all the conflicts, to all society members engaged in a specific conflict, or to all the costs that a specific society encounters (Ditlmann et al., 2017). There is no doubt that, in every society, there are costs that are greatly valued, at least by a segment of society, and therefore are being eliminated. For example, a study by Gayer et al. (2009) carried out in Israel found that exposure to information about specific costs that would be the result of the continuation of the conflict led individuals to increased openness to alternative information and support for conciliatory policies to promote a peaceful conflict resolution. These costs are related to the disappearance of the Jewish identity of the Israeli state or the deterioration of the economy. However, in general, the way of providing information about costs that a society pays for the continuation of a bloody and lasting conflict, as a method of moderating views and promoting conflict resolution, has not been receiving much attention. Therefore, in this research, we explore what kind of costs, and under what conditions, exposure to major costs of an intractable conflict affects openness to information and conflict-related attitudes among Israeli Jewish society members.

### Intractable Conflicts and Their Costs

Harsh and protracted inter-group conflicts, referred to as intractable conflicts, are distinguished by their violence, duration and intensity, underlying causes, and the toll they take on the involved societies (Kriesberg, 2005; Kelman, 2007; Marcus, 2014). In many cases, they engage rivals with significant power asymmetries, as in the Israeli–Palestinian conflict (Maoz and McCauley, 2008; Kriesberg, 2009)^1^. One of the characteristics of intractable conflicts is that they demand extensive investment. As these conflicts tend to be locked in a vicious circle of escalation and de-escalation characterized by chronic violence (Brubaker and Laitin, 1998; Lake and Rothchild, 1998; Kelman, 2007; Rosler et al., 2017), the investment they require can be alternatively viewed as costs. The many lives lost and suffering from physical injuries during the conflict are probably the ultimate ones. However, beyond these tremendous costs, societies involved in a prolonged violent conflict pay other grave costs, out of which, in the current research, we focus on three that stand out: costs related to mental health, economy, and democratic principles (Bar-Tal and Raviv, 2021). We selected these three costs because in previous studies it was found that the major cost of losses of life is generally discounted in Israel (Kanetti Excel, 2021; Shavit-Caftori, 2021). That is, since the frequent violence is mostly encountered by soldiers, it is viewed by Israeli Jews as a necessary sacrifice to ensure the existence of the state (Hirschberger et al., 2015).

Prolonged exposure to violence, either by experiencing or perpetuating it, increases mental health costs. It entails severe negative psychological effects on the entire population, manifested by continuous emotional and physiological arousal of symptoms, such as chronic sense of anxiety, fear and insecurity, ongoing stress, and post-traumatic stress disorder (PTSD) (Bar-Tal, 2001; Canetti-Nisim et al., 2009; Gallagher et al., 2012; Rieder and Elbert, 2013; Rosshandler et al., 2016). Psychological distress may negatively affect not only the direct victims and perpetuators of violence but also bystanders, relatives, or even people who are vicariously exposed to it through the media (Schuster et al., 2001; Bleich et al., 2003; Hobfoll et al., 2006). More specifically, security and military forces injured and/or exposed to violence are direct primary victims of serious harm to mental health. In Israel, Bleich et al. (2003) estimated that a high fraction of Israelis, over one-third, suffer from trauma- and stress-related mental health symptoms.

Protracted conflicts also entail a heavy economic burden on involved societies and states. States in an intractable conflict have to allocate huge resources to maintain their security forces aimed at defending their existence and preventing physical violence and compensate victims and their relatives of life and property damages (Lifshitz, 1998). Such states may suffer from various economic sanctions and boycotts initiated and promoted by their rivals (Grossman et al., 2018) while losing the potential economic growth that ending the conflict peacefully may enable (e.g., Appel and Loyle, 2012). In their essence, the economic costs of the conflict shift expenditures to military and security matters, significantly decreasing investments in education, health welfare, infrastructure, and so on. In Israel, these costs result in the undermining of economic growth, rising inequality, and poverty (Swirski and Dagan-Buzaglo, 2017).

Another major cost of prolonged conflicts is the deterioration of principles of human rights and democracy. While liberal democracies are based on basic liberties, such as freedom of expression, free flow of information, and security of minority rights, the effective management of intractable conflicts requires limiting or, in extreme cases, even abolishing those liberties (Rosler et al., 2009). Justified mainly by the need to provide security for a society and its members who are under constant threat by the conflict, blocking information, monitoring minorities, and oppressing opposition groups who challenge conflict-related policies become acceptable and are sometimes considered as necessary measures (Arian, 1995; Shahar et al., 2018; Harel et al., 2020). In Israel, these processes have accelerated in recent years, as the government is making efforts to disseminate hegemonic conflict-supporting narratives *via* institutions, organizations, mass media, and educational system, while at the same time trying to suppress the flow of information that contradicts these narratives (Oren et al., 2015).

It is clear that the social agents and institutions of societies engaged in a bloody and lasting conflict make an effort to hide the costs and prevent public debate about them. One can argue that exposure to the immense costs that intractable conflicts require and public discourse about them could diminish considerably the readiness to mobilize for the conflict and even the willingness to sacrifice life. Thus, societies that aspire to achieve maximal goals in the violent conflict need to develop rationalizations and justifications for the continuation of the conflict that will prevent questioning of the costs (Bar-Tal, 2013; Adisonmez, 2019).

The most common way to cope with this challenge is to develop and then maintain functional conflict-supporting collective narratives that fulfill this goal. A few pillars in these narratives are very relevant to the justification of the costs. The first one is the theme of justness of goals, which is presented as being of supreme importance and of existential nature and provides a meaningful and coherent rationale to why major sacrifices have to be made. The second pillar is narratives about security and refers to the mobilization and material investment in the conflict that are necessary to achieve this basic goal. Finally, the third pillar is narratives about patriotism, with its glorified ultimate symbol of devotion and willingness to sacrifice for the collective during the conflict (Eidelson and Eidelson, 2003; Hadjipavlou, 2007; Papadakis, 2008; Hammack, 2009; Garagozov, 2012; Bar-Tal, 2013). Due to their great functionality in dealing with the challenges of the conflict and, more specifically, its severe costs, conflict narratives often become hegemonic, widely shared by society members and deeply entrenched (Maoz and McCauley, 2008; Halperin and Bar-Tal, 2011; Vollhardt and Bilali, 2015; Rosler et al., 2018). However, they also feed the continuation of the conflict by providing rationale for the cycles of violence and their costs and block the flow of crucial information for resolving the conflict peacefully and the openness of society members to such information (Halperin and Bar-Tal, 2011).

### This Research

In this research, we examined whether, under what conditions, and for whom messages about costs that are associated with the continuation of the conflict will lead individuals to be more open to alternative information about the conflict and more supportive of conciliatory policies. In the messages, we highlighted some of the most difficult current and future costs of the Israeli–Palestinian conflict, which serves as a typical intractable conflict (e.g., Cohen, 2005). As noted, we decided to focus on three major costs of the ongoing conflict for the Jewish Israeli society: the mental health cost, economic cost, and the cost for democratic norms, which often appear in a public discourse in Israel. We believe these three costs provide a strong contest for the existing conflict-supporting narrative. Hence, if Israeli-Jews are exposed to them, and are willing to thoroughly process them, they can potentially create attitude change. Conversely, discovering their inability to induce such change would provide further support for the strength of the conflict narratives as adamant barriers to conflict resolution (see Halperin and Bar-Tal, 2011; Porat et al., 2015). The two studies were carried out in June 2020, with the Israeli–Palestinian conflict in a prolonged stalemate situation and the COVID-19 pandemic affecting people around the world.

## Study 1

In Study 1, we tested the effect of the current costs of the conflict on attitudes regarding continuation and peaceful resolution of the conflict. We examined whether an intervention based on messages providing information from a reliable source on each of the costs (i.e., mental health and economic costs of the conflict, and costs to Israeli democracy) would lead to unfreezing of conflict-related attitudes manifested by more openness to alternative information, and increased support for peaceful policies on the conflict. We further investigated if the predispositions of the participants acknowledging the specific costs that Israel pays for the continuation of the conflict would be associated with openness to alternative information and support for peaceful policies.

### Method

#### Participants

Two hundred and fifty-five Jewish Israeli participants (*M*_age_ = 40.36, *SD*_age_ = 14.71; 125 men, 130 women; 43.1% were secular, 36.1% considered themselves as observant, 11% were religious, and 9.8% were ultra-orthodox; in terms of political orientation, 8.6% were strong rightists, 23.1% were rightists, 25.5% were moderate rightists, 22% were centrists, 13.3% were moderate leftists, 6.7% were leftists, and 0.8% were strong leftists) completed the questionnaires through the online surveying firm iPanel, which is an opt-in panel that includes over 100,000 panelists within Israel. The sample resembled the adult Jewish Israeli population (Hermann et al., 2018; Central Bureau of Statistics, 2020). In exchange for participation, the participants received 4.5 ILS (equivalent to US$1.4). Power calculations indicated that the sample size was big enough to detect with 0.8 power a medium effect size (Cohen's *f* = 0.21) for the condition main effect.

#### Procedure

The participants were asked to take part in a study in which they would read a short text and respond to some questions. They were then randomly assigned to one of four conditions. Participants in the mental health condition (*n* = 64), economic condition (*n* = 64), and democracy condition (*n* = 63) were asked to read a short text in Hebrew, informing them about a study carried out by an independent American research institution that points to the current mental health, economic, or democracy costs that Israel pays for the Israeli–Palestinian conflict. In the control condition, participants (*n* = 64) read a text about a research study that focused on the costs of polluting the seas of Israel that have nothing to do with the Israeli–Palestinian conflict.

After reading the texts, participants were asked to answer two attention verification questions. The participants who answered these questions correctly continued to complete the dependent variables questionnaire, which included the measures detailed below and some additional exploratory measures (for complete materials and data for Studies 1 and 2, see https://osf.io/xmdpt/?view_only=9c1bf0f996484ed2976411f8391346af).

#### Measures

Unless indicated otherwise, all items were measured using a scale from 1 = not at all to 6 = to a great extent.

Perception of costs. Five items assessed the perceptions of the participants about the extent (from 1 = very detrimental to 6 = very beneficial) to which the continuation of the conflict is detrimental or beneficial to the state of Israel in general and with regard to the mental health of Israelis, the economy of Israel, budgets for welfare, and the democracy of Israel.

Openness to alternative information was measured with three items (α = 0.86) assessing the willingness of the participants to (a) be exposed to Palestinian movies that convey the Palestinian perspective of the conflict; (b) personally meet Palestinians and hear their views about the conflict; and (c) be exposed to critical information about the manner by which the Israeli government is managing the conflict (see Halperin and Bar-Tal, 2011; Hameiri et al., 2018).

Support for negotiations and conciliatory policies was measured using five items (α = 0.83) assessing the support of the participants (from 1 = completely oppose to 6 = completely support) for negotiations to obtain different outcomes (i.e., achieving peace between Israelis and Palestinians, long-term truce between Hamas and Israel, and establishment of a demilitarized Palestinian state in the West Bank) and conciliatory policies (i.e., freezing construction of Israeli settlements in the West Bank and building an airport and a seaport in the Gaza Strip; see Hameiri et al., 2016).

Political orientation was measured with a standard self-identifying item for assessing political orientation with regard to security-related issues and the Israeli–Palestinian conflict on a scale ranging from 1 = strong right to 7 = strong left.

### Results

For means, SDs, and correlations across all measured variables, see Table 1. To examine the effect of the intervention, we ran a series of one-way ANOVAs for each of the dependent variables (see means and SDs for each condition in Table 2). Since the conditions were unbalanced in terms of religiosity (with the participants in the democratic cost condition significantly more secular than those in all other conditions; all *p*s < 0.044) and gender [with 53.1 women to 46.9% men in the mental health cost condition, 45.3 women to 54.7% men in the economic cost condition, 65.1 women to 34.9% men in the democracy cost condition, and 40.6 women to 59.4% men in the control condition; χ^2^(3) = 8.7, *p* = 0.034; conditions were similar in terms of political orientation and age of the participants; all pairwise comparison *p*s > 0.530], we controlled for these background variables throughout the statistical analysis in order to eliminate potential alternative explanations. Not controlling these variables had no effect on the results (see Supplementary Materials).

**Table 1:** Means, SDs, and bivariate correlations among Study 1 variables.

	**1**	**2**	**3**	**4**	**5**	**6**	**7**	**8**	**9**	**10**	**11**
M	3.46	3.5	1.87	2.29	2.22	2.57	2.12	2.08	3.31	40.36	–
SD	1.17	1.34	0.91	1.03	1.04	1.12	1	1.32	1.4	14.71	–
1. Support for negotiations and conciliatory policies	–										
2. Openness to alternative information	0.54^**^	–									
3. Conflict cost: general	−0.24^**^	−0.22^**^	–								
4. Conflict cost: economy	−0.15^**^	−0.12	0.46^**^	–							
5. Conflict cost: welfare	−0.3^**^	−0.28^**^	0.43^**^	0.63^**^	–						
6. Conflict cost: democracy	−0.25^**^	−0.25^**^	0.45^**^	0.46^**^	0.53^**^	–					
7. Conflict cost: mental health	−0.19^**^	−0.18^**^	0.41^**^	0.44^**^	0.49^**^	0.53^**^	–				
8. Religiosity	−0.43^**^	−0.21^**^	0.2^**^	0.14^*^	0.22^**^	0.17^**^	0.13^*^	–			
9. Political orientation	0.72^**^	0.51^**^	−0.22^**^	−0.21^**^	−0.33^**^	−0.27^**^	−0.24^**^	−0.44^**^	–		
10. Age	0.25^**^	0.07	0.01	−0.03	−0.02	0.07	0.13^*^	−0.03	0.17^**^	–	
11. Gender	−0.05	−0.11	−0.17^**^	−0.09	−0.09	−0.16^**^	−0.2^**^	−0.04	−0.004	−0.22^**^	–

* *p < 0.05;*

** *p < 0.01*.

**Table 2:** Means and comparisons of each condition to control across all DVs of Study 1.

**Condition**	**Conflict cost: mental health**	**Conflict cost: democracy**	**Conflict cost: welfare**	**Conflict cost: economy**	**Conflict cost: general**	**Openness to alternative information**	**Support for negotiations and conciliatory policies**
Control	2.11 (1.01)	2.69 (1.26)	2.28 (0.97)	2.41 (1.08)	2.02 (1.1)	3.56 (1.47)	3.61 (1.29)
Cost to mental health	2.12 (1.13)	2.6 (1.18)	2.24 (1.07)	2.41 (1.1)	1.74 (0.76)	3.6 (1.31)	3.51 (1.18)
Cost to economy	2.16 (1.01)	2.47 (1.05)	2.13 (1.22)	2.02 (0.98)^*^	1.79 (0.9)	3.51 (1.31)	3.48 (1.14)
Cost to democracy	2.11 (0.86)	2.53 (0.98)	2.25 (0.9)	2.34 (0.92)	1.95 (0.86)	3.32 (1.28)	3.23 (1.05)^†^

† *p < 0.10;*

* *p < 0.05*.

The one-way ANOVAs yielded a marginally significant main effect [*F*_(3,249)_ = 2.2, *p* = 0.088, partial η^2^ = 0.03] of the experimental condition on whether the conflict is perceived to be detrimental or beneficial to the Israeli economy. Paired comparisons revealed that the economic cost condition led participants to perceive the Israeli–Palestinian conflict as significantly more detrimental to Israel compared with the mental health cost and control conditions (both *p*s < 0.03) and marginally more compared to the democracy cost condition (*p* = 0.083). No significant differences were found between conditions on the remaining four items that assess perceived costs, openness to alternative information, and support for negotiations and conciliatory policies (all *p*s > 0.232).

Correlation Analysis. Our manipulation highlighting different costs the Israeli–Palestinian conflict has on Israel and the Israeli society had almost no significant effect. Thus, we examined whether the held perceptions about whether the continuation of the conflict is detrimental or beneficial to the mental health of Israelis, economy of Israel, and democracy (i.e., whether they are perceived as costs) predicted openness to alternative information and support for negotiations and conciliatory policies. We examined this with two linear regression models in which, on top of the demographic variables we controlled throughout the statistical analysis, we also controlled for condition (practically, we controlled for three dummy variables that reflected the comparisons between each of the cost conditions and the control condition). We found that the perception about the cost to democracy was a single significant predictor for both openness to alternative information (*b* = −0.25, *SE* = 0.09, *t* = −2.84, *p* = 0.005) and support for negotiations and conciliatory policies (*b* = −0.19, *SE* = 0.07, *t* = −2.63, *p* = 0.009), which were not significantly predicted by both perceptions about the costs to the mental health of Israelis and to the economy of Israel (all *p*s > 0.218).

### Discussion

The results of the first study revealed that the intervention, based on providing information about one of three sets of grave costs that Israel is paying for the continuation of the conflict, had (almost) no significant effect on the perceptions of the participants of these prices or openness to new information about the conflict. Accordingly, it also had no impact on the support of Jewish Israelis for negotiation and conciliatory policies on the conflict. Therefore, these results could reflect the power of the entrenched conflict-supporting narratives that justify the required investments for pursuing the conflict goals while presenting those sacrifices as patriotic devotion. They also showed the power of routinization, indicating that Israeli Jews view the lasting life under the bloody conflict as being normal with its costs as a necessary part of life. Living for over 70 years with the conflict made Israeli Jews accustomed to the cost-full life, and they think that an alternative provides a riskier, uncertain, and insecure future (Mitzen, 2006; Marcus, 2014; Rumelili, 2014; Elman et al., 2019; Kossowska et al., 2020; Bar-Tal and Raviv, 2021).

Interestingly, the perceptions that the participants had about the cost to Israeli democracy (but not to mental health or the economy) were the sole significant predictor for both openness to new information and support for negotiation and conciliatory policies on the conflict. In light of this result and past studies that pointed to the positive effect of information about a prospective solution to the conflict that threatens the Jewish character of Israel on openness to new information and conciliatory attitudes (Gayer et al., 2009), we decided to examine a second intervention. Hence, the second study examined whether information about future potential costs to the democracy of Israel or to its Jewish character created by the continuation of the conflict will create attitude change regarding the conflict.

## Study 2

The goal of Study 2 was to examine the effect of future costs to the Jewish or democratic character of Israel due to the continuation of the conflict on attitudes regarding its resolution. The tension and potential contradiction between the Jewish and democratic nature of the country have been a prominent issue in its social and political landscapes for decades (e.g., Shafir and Peled, 2002). While Study 1 pointed to the association between existing perceptions concerning democracy and conflict-related attitudes in Israel, Gayer et al. (2009) found that information relating to its Jewish character affects these attitudes. Therefore, Study 2 examined the effect of information about future cost to each of the two principles on conflict-related attitudes.

Past studies that examined the balance between the two main values that relate to the identity of Israel as a Jewish democratic state indicated different preferences according to the level of religiosity of a respondent (Shamir and Shamir, 2000). The Jewish Israeli society comprises communities differing in the level of adherence to the main beliefs and practices of Judaism. While the largest segment is composed of seculars who usually practice only few religious commandments, a very similar segment in size is composed of traditional or observant Jews who maintain some Jewish customs and religious duties. The two smaller groups are religious and ultra-Orthodox who both adhere to Jewish practices but differ in level of integration in the civic life in Israel, with the latter being mostly committed to segregation (Okun, 2017). A recent survey clearly showed that while the majority of secular Israeli Jews prefer the democratic identity, most religious and ultra-orthodox Israelis prefer the Jewish identity (Hermann et al., 2018). Therefore, we hypothesize that the effect of an intervention based on messages providing information from a reliable source on the potential future cost of conflict maintenance to the Jewish or democratic identity of Israel will vary according to religiosity. We expect that levels of religiosity will moderate the effect of the intervention on openness to new information and on support for negotiation and conciliatory attitudes. Specifically, we expect that future costs to the democratic identity of Israel will be more effective among participants with lower levels of religiosity; whereas future costs to the Jewish identity of Israel will be more effective among those with higher levels of religiosity.

### Method

#### Participants

Two hundred and fifty-five Jewish Israeli participants (*M*_age_ = 40.56, *SD*_age_ = 14.64; 123 men, 132 women; 43.9% were secular, 34.5% considered themselves as observant, 11.4% were religious, and 10.2% were ultra-orthodox; in terms of political orientation, 6.3% were strong rightists, 34.1% were rightists, 20% were moderate rightists, 23.5% were centrists, 12.2% were moderate leftists, 2.7% were leftists, and 1.2% were strong leftists) completed the questionnaires through the online surveying firm iPanel. The sample resembled the adult Jewish Israeli population (Hermann et al., 2018; Central Bureau of Statistics, 2020). In exchange for participation, the participants received 4.5 ILS (equivalent to US$1.4). Power calculations indicated that our sample size was big enough to detect with 0.8 power and a medium effect size (Cohen's *f* = 0.20) for the condition main effect.

#### Procedure and Materials

The participants were asked to take part in a study in which they would read a short text and respond to some questions. They were then randomly assigned to one of three conditions. Participants in the Jewish identity (*n* = 85) and democracy conditions (*n* = 85) were asked to read a short text in Hebrew, informing them about a position paper written by the US National Security Council that points to the future cost to the Jewish identity of Israel or to its democratic character, if the Israeli–Palestinian conflict is maintained without a peaceful resolution. In the control condition, participants (*n* = 85) read a text about a position paper on an energy plan for the US that has nothing to do with the Israeli–Palestinian conflict.

After reading the texts, participants were asked to answer two attention verification questions. The participants who answered these questions correctly continued to complete the dependent variables questionnaire, which included the measures detailed below and some additional exploratory measures. We measured openness to alternative information (α = 0.82) and support for negotiations and conciliatory policies (α = 0.80) with the exact same items in Study 1. Since this study focused on future costs of the conflict, we did not include the same items that served to test perceptions of current costs in Study 1.

#### Results

For means, SDs, and correlations across all measured variables, see Table 3. Since our conditions were unbalanced in terms of age (with the participants in the Jewish identity cost condition significantly older than those in the control condition; *p* = 0.012) and gender (with 63.5 women to 36.5% men in the democracy cost condition, 49.4 women to 50.6% men in the Jewish identity cost condition, and 42.4 women to 57.6% men in the control condition; χ^2^(2) = 7.92, *p* = 0.019; conditions were similar in terms of political orientation of the participants; all pairwise comparison *p*s > 0.201), we controlled these background variables throughout the statistical analysis in order to eliminate potential alternative explanations. Not controlling these variables had no effect on the results (see Supplementary Materials). To examine the effects of the manipulation moderated by levels of religiosity of the participants, we used the PROCESS macro (model 1) of Hayes (2018) with 5,000 bootstrap re-samples for a multi-categorical independent variable using indicator coding (Hayes and Montoya, 2017).

**Table 3:** Means, SDs, and bivariate correlations among Study 2 variables.

	**1**	**2**	**3**	**4**	**5**	**6**
M	3.4	3.4	2.09	3.14	40.56	–
SD	1.05	1.31	1.35	1.32	14.64	–
1. Support for negotiations and conciliatory policies	–					
2. Openness to alternative information	0.44^**^	–				
3. Religiosity	−0.46^**^	−0.14^*^	–			
4. Political orientation	0.61^**^	0.37^**^	−0.33^**^	–		
5. Age	0.21^**^	−0.004	−0.14^*^	0.16^*^	–	
6. Gender	0.02	−0.17^**^	0.07	−0.06	0.01	–

* *p < 0.05;*

** *p < 0.01*.

**Figure 1:**
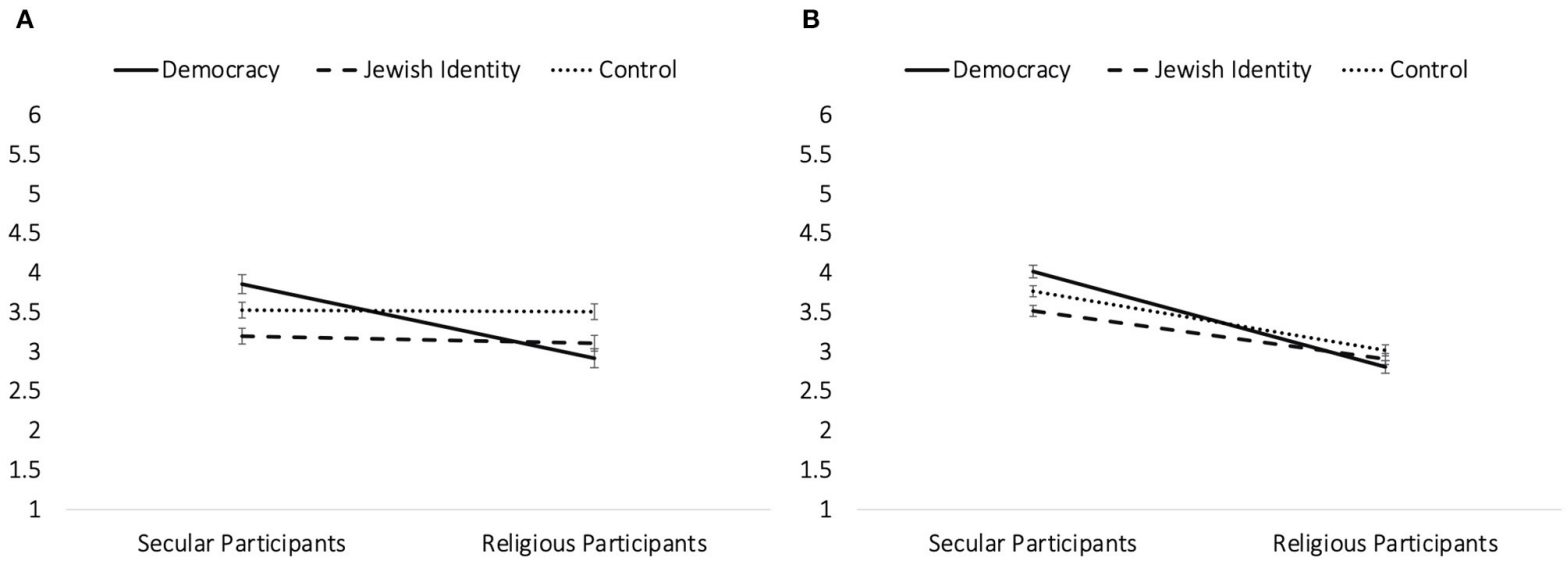
The results of the interaction between the condition and level of religiosity of participants on **(A)** openness to alternative information and **(B)** support for negotiations and conciliatory policies in Study 2. Error bars represent SEs.

Openness to alternative information. Levels of openness to alternative information of the participants were marginally significantly lower in the cost to Jewish identity condition (*M* = 3.16) compared with the control (*M* = 3.52; *b* = −0.36, *SE* = 0.2, *t* = −1.82, *p* = 0.07; 95% CI = [−0.75,0.03]), while all other comparisons between these conditions and the cost to democracy condition (*M* = 3.44) were not significant (both *p*s > 0.168). Importantly, we also found a significant condition × religiosity interaction [*F*(_2,247)_ = 3.63, *p* = 0.028, *R*^2^ change = 0.027; see Figure 1A]. Conditional effects revealed that, for the more secular participants, the cost to democracy condition led to more openness to alternative information (*M* = 3.86) compared with the cost to Jewish identity condition (*M* = 3.2; *b* = 0.66, *SE* = 0.25, *t* = 2.61, *p* = 0.01; 95% CI = [0.16, 1.15]), while both conditions did not significantly differ from the control condition (*M* = 3.53; both *p*s > 0.2). However, for the more religious participants, the cost to democracy condition led to less openness to alternative information (*M* = 2.92) compared with the control condition (*M* = 3.51; *b* = −0.59, *SE* =0.29, *t* = −2.03, *p* = 0.044; 95% CI = [−1.17, −0.02]), while both conditions did not significantly differ from the cost to Jewish identity condition (*M* = 3.11; both *p*s > 0.128).

Support for negotiations and conciliatory policies. Levels of support of the participants for negotiations were marginally significantly predicted by the condition × religiosity interaction [*F*_(2,247)_ = 2.71, *p* = 0.068, *R*^2^ change = 0.016; see Figure 1B]. For the more secular participants, conditional effects revealed that the cost to democracy condition led to more support for negotiations and conciliatory policies (*M* = 4.02) compared with the cost to Jewish identity condition (*M* = 3.52; *b* = 0.5, *SE* = 0.25, *t* = 2.76, *p* = 0.006; 95% CI = [0.14, 0.86]), while both conditions did not significantly differ from the control condition (*M* = 3.77; both *p*s > 0.176). However, for the more religious participants, support for negotiations and conciliatory policies was similar across the three conditions (all *p*s > 0.309).

## General Discussion

Intractable conflicts require a great investment from the involved societies. Conflict-supporting narratives, developed to functionally cope with the challenges the conflicts create, provide rationale for this investment as necessary and justified. However, such perceptions regarding the investment in a conflict block the path to reconsider it as a heavy and potentially avoidable cost and thus serve as a sociopsychological barrier to peacefully resolving the conflict (Halperin and Bar-Tal, 2011). In the two studies, we examined whether providing Israeli Jews with information about major costs paid by their society for the continuation of the protracted Israeli–Palestinian conflict could create attitude change toward considering new information and supporting peace. In the first study, we found that providing such information about three major current costs, concerning the mental health of the citizens of Israel, its economy, and democracy, does not create openness to new information and support for its peaceful resolution. However, predispositions regarding the cost to the democracy of Israel were positively associated with increased openness and support for peace. In the second study, we discovered that providing information on the future cost of continuing the conflict to the democratic identity of Israel significantly affected the attitudes regarding the conflict, while the effect was moderated by the level of religiosity. For secular participants, this manipulation created more openness to alternative information about the conflict and increased support for conciliatory policies, but for religious participants, that same manipulation backfired, creating less openness to alternative information. It should be noted that these results were obtained during the difficult context of the COVID-19 pandemic, although Israel was doing relatively well at that point of time.

### Theoretical and Applied Implications

This study provides partial support for the role of information about losses in advancing the resolution of intractable conflicts while pointing to its limitations in this context. The asymmetry between information about gains and information about losses, with the latter being more influential, has been suggested by the prospect theory (Kahneman and Tversky, 1979). This is due to the fact that human beings are more reluctant to lose what they already have than to gain something that they still do not have when the objects and commodities are interchangeable and hence can be framed as losses or gains. Previous studies have found a negativity bias showing that negative information and events are dominant in human attention, memory, judgment, and decision-making compared to positive ones (Rozin and Royzman, 2001; Soroka et al., 2019). When dealing specifically with conflicts, Gayer et al. (2009) have suggested that framing the continuation negatively with concrete and valid loss arguments increases willingness to be exposed to different information and support concessions. However, the study specifies more accurately which arguments could work better to which audiences (see Bar-Tal and Hameiri, 2020; Halperin and Schori-Eyal, 2020). While for non-religious Israelis concrete future threat to one of their major political values of democracy could create attitude change in the context of a violent and prolonged conflict, a similar message has no influence on religious society members. It seems that religious Israelis, similar to rightist Israelis (Hermann and Yuchtman-Yaar, 2002; Gayer et al., 2009), adhere to a larger extent to the rigid conflict-supporting narratives, therefore making them less receptive to the negative framing of the continuation of conflict as leading to a real future threat to the Jewish identity of Israel. Furthermore, framing it as a potential threat to the democratic identity of Israel backfires, possibly since demoting this aspect of the identity of Israel could conform with their values to some extent (Liebman, 1997; Shamir and Shamir, 2000; Ben-Rafael, 2008; Hermann et al., 2018).

Moreover, the study highlights the basis for the major schism between the religious and secular segments of the society. While other investigated costs harm more or less equally the entire Jewish population of Israel, the costs of the potential loss of the democratic pillar of the state are especially valid for the secular population and have become, as a result, very salient in this population. This is evident in public discourse and struggle in the political and civil arenas. Moreover, the challenge of maintaining the democratic nature of Israel has, in recent years, become one of the key issues in Israel (Waxman and Peleg, 2020; Bar-Tal and Raviv, 2021). Unilateral coercive strategies to manage the Israeli–Palestinian conflict have been brought to the foreground by major political actors in Israel and, as a result, have created growing awareness to the harm they may cause to the democratic nature of the country. Partial annexation of the West Bank, for example, will most likely lead to the collapse of the Palestinian National Authority, forcing Israel to create an apartheid-like authoritarian regime over the Palestinian population residing in the West Bank (Sher and Cohen, 2019). Others have suggested that such harmful outcomes for democracy are a direct result of the prolonged occupation that Israel maintains (Rosler et al., 2009). In both cases, in order for costs to influence public views, they have to be noticeable and openly discussed. The public discourse about the possibility of annexation, together with the current political struggle in Israel regarding corruption and its implications on democratic norms and institutions, turned the cost into an issue that is commonly addressed and cannot be hidden anymore (Bar-Tal and Raviv, 2021). Therefore, we suggest that awareness of this cost affects the attitudes of some society members while causing the alienation of others, as shown by this study.

In addition, our findings contribute to the discussion in the literature regarding the relationship between religiosity, democratic values, and conflict-related attitudes. Past studies have generally indicated a somewhat complex relationship between religiosity and support for democracy (Ben-Nun Bloom and Arikan, 2012) and a negative association of religiosity and support for democracy in Israel mediated by authoritarianism (Canetti-Nisim, 2004). However, the negative association between religiosity and support for negotiation and conciliatory policies is well-established (e.g., Hermann and Yuchtman-Yaar, 2002; Maoz and McCauley, 2009; Shamir and Shikaki, 2010). The findings add another layer of understanding to the role of religiosity concerning democracy and conflict by indicating that presenting future costs to the democratic and Jewish identities of Israel actually increases the freezing of conflict-related attitudes of a religious individual. Religious participants not only rejected the idea that the continuation of the conflict may threaten the religious identity of Israel but also evidenced lower levels of openness to alternative information about the conflict when confronted with the potential cost to democracy. Therefore, we can suggest that religiosity and conflict-supporting narratives heighten the barriers to resolving the conflict by refusing to acknowledge the costs of conflict and, thus, open up to peaceful alternatives.

The findings also highlight the lack of interest and the denial practiced by the Jewish Israeli society, a society engulfed by an intractable conflict, to the grave mental health and economic costs of maintaining the bloody conflict in which it has been involved for over a century. Living in such a context created a need to develop a strong patriotism that requires ongoing mobilization and routinization of ways of life, enabling coping with the challenges that pose lasting bloody confrontations. Societies that live under the shadow of intractable conflicts are accustomed to sacrificing lives for their cause, enduring economic hardship, living under chronic fear, and even bearing autocratic regime, all for survival, as presented by the leaders and formal institutions that construct rationalizing narratives. Routinization of the conflict, in spite of potential minorities who may deviate from mainstream thoughts, is becoming a way of life. Thus, routinization is one of the factors responsible for not seeing all the costs and the normalized way of life that is necessary in view of existential threats (Bar-Tal et al., 2014).

Finally, the leaders of a society and other formal social institutions prevent the free flow of information and debates. They try to thwart messages that contradict the hegemonic narratives they propagate (Oren et al., 2015). For example, the Minister of Education issued a decree that prevents non-government organizations (NGOs) that collect information about the violation of human rights in the West Bank to enter schools. Thus, free debates about the costs are discouraged, and the majority of the mass media follow this course (see Bar-Tal and Raviv, 2021 for review). As a result, societies involved in an intractable conflict might give way to obedience, conformity, and self-censorship. Most society members who are aware of the costs incurred prefer not to harm the society by the opened discussion and/or are afraid of the negative sanctions that may be used by the institutions or even their social circles for expressing criticism of the way the society goes (Bar-Tal et al., 2017). Eventually, by freezing with their hegemonic narratives that justify the conflict, its continuation itself becomes a necessary part of the ontological security of the state (Mitzen, 2006; Rumelili, 2014; Bar-Tal and Raviv, 2021), with fear to take even a small risk to change the bloody context. Nevertheless, when the cost becomes a key social issue, raising the awareness of individuals in the context of the conflict can make a difference. This is an optimistic message of this study.

At this point, it should be noted that, due to budgetary constraints, this study was relatively underpowered. While the sample sizes were sufficiently large to detect a small to medium effect size for the condition main effect, this study was relatively underpowered to detect the interaction between the condition and religiosity of the participants in Study 2. This may have resulted in the fact that one of the interactions in Study 2 was only marginally significant and thus should be interpreted with due caution. To summarize, although intractable conflicts impose tremendous costs over societies involved in them, most of the society members remain closed to alternative information that can serve the goal of resolving intractable conflicts peacefully. Examining the prototypical Israeli–Palestinian conflict, this study reveals which arguments about losses could work better on specific groups within society. By doing so, it suggests a potential application that could be further developed in order to create an opening for a change in these difficult conflicts.

## Data Availability Statement

The raw data supporting the conclusions of this article is available on https://osf.io/xmdpt/?view_only=9c1bf0f996484ed2976411f8391346af.

## Ethics Statement

The studies involving human participants were reviewed and approved by Tel Aviv University ethics committee. The patients/participants provided their written informed consent to participate in this study.

## Author Contributions

NR, DC, and SA-T organized the database. BH performed the statistical analysis. NR and BH wrote the first draft of the manuscript. NR, BH, and DB-T wrote sections of the manuscript. All authors contributed to conception, design of the study, manuscript revision, read, and approved the submitted version.

## Conflict of Interest

The authors declare that the research was conducted in the absence of any commercial or financial relationships that could be construed as a potential conflict of interest.

## Footnotes

^1^While the costs they create for the high power vs. the low power groups could be somewhat different, discussing those variations is beyond the scope of this study.
