# Supplementary material for: Current and Future Costs of Intractable Conflicts—Can They Create Attitude Change?
Source: Front Psychol. 2021 May 26;12:681883. doi: 10.3389/fpsyg.2021.681883 (PMC8187953; doi:10.3389/fpsyg.2021.681883)
Supplement: Supplementary file 1 [file Data_Sheet_1.docx]

**Current and Future Costs of Intractable Conflicts – Can They Create Attitude Change?**

**Supplementary Materials**

*Additional analysis without controlling for participants’ religiosity and gender: Study 1*

To examine the effect of our intervention, we ran a series of one-way ANOVAs for each of our dependent variables. The one-way ANOVAs yielded no significant differences between conditions on the five items that assess perceived costs, openness to alternative information, and support for negotiations and conciliatory policies (all *p*s > .112).

*Additional analysis without controlling for participants’ religiosity and gender: Study 2*

To examine the effects of the manipulation moderated by participants’ levels of religiosity, we used Hayes’s (2018) PROCESS macro (Model 1) with 5,000 bootstrap resamples for a multicategorical independent variable by using indicator coding (Hayes & Montoya, 2017).

*Openness to alternative information.* Levels of participants’ openness to alternative information was marginally significantly lower in the cost to Jewish identity condition (*M* = 3.17) compared to the control (*M* = 3.58; *b* = -.41, *SE* = .20, *t* = -2.05, *p* = .042; 95% CI = [-.80, -.02]), while all other comparisons between these conditions and the cost to democracy condition (*M* = 3.38) were not significant (both *p*s > .285). Importantly, we also found a marginally significant condition × religiosity interaction (*F*(2, 249) = 2.82, *p* = .062, *R^2^* change = .021). Conditional effects revealed for the more secular participants, the cost to democracy condition led to more openness to alternative information (*M* = 3.79) compared to the cost to Jewish identity condition (*M* = 3.22; *b* = .57, *SE* = .25, *t* = 2.24, *p* = .026; 95% CI = [.07, 1.07]), while both conditions did not significantly differ from the control condition (*M* = 3.63; both *p*s > .116). For the more religious participants, however, the cost to democracy condition led to *less* openness to alternative information (*M* = 2.89) compared to the control condition (*M* = 3.51; *b* = -.62, *SE* = .30, *t* = -2.10, *p* = .037; 95% CI = [-1.21, -.04]), while both conditions did not significantly differ from the cost to Jewish identity condition (*M* = 3.11; both *p*s > .131).

*Support for negotiations and conciliatory policies.* Levels of participants’ support for negotiations was significantly predicted by the condition × religiosity interaction (*F*(2, 249) = 3.38, *p* = .036, *R^2^* change = .021). Conditional effects revealed for the more secular participants, the cost to democracy condition led to more support for negotiations and conciliatory policies (*M* = 4.07) compared to the cost to Jewish identity condition (*M* = 3.55; *b* = .50, *SE* = .18, *t* = 2.77, *p* = .006; 95% CI = [.14, .86]), and marginally more compared to the control (*M* = 3.74; *b* = .32, *SE* = .18, *t* = 1.77, *p* = .078; 95% CI = [-.04, .67]). The difference between the cost to Jewish identity condition and control was not significant (*p* = .328). For the more religious participants, however, support for negotiations and conciliatory policies was similar across the three conditions (all *p*s > .310).

**References**

Hayes, A. F. (2018). *Introduction to mediation, moderation, and conditional process analysis: A regression-based approach* (2nd ed.) The Guilford Press.

Hayes, A. F., & Montoya, A. K. (2017). A tutorial on testing, visualizing, and probing an interaction involving a multicategorical variable in linear regression analysis. *Communication Methods and Measures*, *11*(1), 1–30.
